# Measured intrapatient radiomic variability as a predictor of treatment response in multi-metastatic soft tissue sarcoma patients

**DOI:** 10.1038/s41598-025-12451-3

**Published:** 2025-07-30

**Authors:** Caryn Geady, James J. Bannon, Shagheyegh Reza, Laura Madanat-Harjuoja, Denise Reinke, Scott Schuetze, Brian Crompton, Andrew Hope, Benjamin Haibe-Kains

**Affiliations:** 1 Department of Medical Biophysics, University of Toronto, Toronto, ON Canada; 2 Princess Margaret Cancer Research, University Health Network, Toronto, ON Canada; 3 Children’s Hospital, University of Helsinki and Helsinki University Hospital, Helsinki, Finland; 4 Finnish Cancer Registry, Helsinki, Finland; 5 Department of Internal Medicine, University of Michigan, Ann Arbor, MI USA; 6 Dana-Farber/Boston Children’s Cancer and Blood Disorders Center, Boston, MA USA; 7 Broad Institute of Harvard and MIT, Cambridge, MA USA; 8 Radiation Medicine Program, Princess Margaret Cancer Centre, University Health Network, Toronto, ON Canada; 9 Department of Radiation Oncology, University of Toronto, Toronto, ON Canada

**Keywords:** Tumor heterogeneity, Radiomics, Liquid biopsy, Treatment response, Cancer, Cancer imaging, Medical imaging, Biomarkers

## Abstract

Radiomics offers a non-invasive approach to tumor characterization, yet its application in metastatic cancers is limited by intertumor heterogeneity—variability in radiomic phenotypes across lesions within the same patient. We introduce Measured Intrapatient Radiomic Variability (MIRV), a novel metric quantifying heterogeneity using standard-of-care imaging. Applied to 397 metastatic soft-tissue sarcoma patients from the SARC021 trial, MIRV was calculated from pretreatment CT scans using pairwise Euclidean distance and cosine dissimilarity between lesions. Euclidean distance captures absolute differences in radiomic features, while cosine dissimilarity assesses variation in feature patterns independent of magnitude. Higher MIRV correlated with greater variability in tumor-specific response classification and volumetric response, independent of baseline tumor volume. In a subset with liquid biopsy data, MIRV showed a moderate association with ctDNA positivity, suggesting links to molecular heterogeneity. While MIRV was not prognostic for overall survival in the full cohort, higher MIRV was significantly associated with worse survival in leiomyosarcoma patients (*n* = 165, *p* = 0.007, FDR = 0.06). These findings establish MIRV as a biomarker for intertumor heterogeneity, with potential to predict mixed treatment responses and guide personalized therapy in metastatic STS. Future studies should assess its relevance across other tumor types and therapeutic settings.

**Supplementary Information:**

The online version contains supplementary material available at 10.1038/s41598-025-12451-3.

## Introduction

Radiological imaging and its quantitative analysis, known as radiomics, is a rapidly evolving field with significant promise for precision oncology. Radiomics enables non-invasive characterization of tumor phenotype and microenvironment^1^, providing insights into spatial heterogeneity that influence prognosis and treatment response in localized cancers^1–3^. However, in the metastatic setting, the primary challenge shifts from intratumoral to intertumoral heterogeneity—the variation in radiomic phenotype across lesions within a single patient. This heterogeneity has critical clinical implications for therapy resistance, disease progression, and mixed treatment response^4–6^.

Prior studies have attempted to characterize lesion-level radiomic features or capture heterogeneity using specialized modalities or tailored machine learning models^3,4,7^. Yet, many of these methods are context-specific and not easily transferable to standard imaging pipelines. To address this gap, we introduce Measured Intrapatient Radiomic Variability (MIRV)—a simple, interpretable, and scalable metric designed to quantify intertumor heterogeneity using conventional CT scans. MIRV summarizes pairwise radiomic dissimilarity across all metastatic lesions within a patient, enabling lesion-agnostic quantification of heterogeneity in a manner broadly applicable across tumor types and clinical settings.

As a proof of concept, we apply MIRV to a large cohort of patients with metastatic soft tissue sarcoma (STS)—a disease marked by extreme clinical, histologic, and molecular heterogeneity^8,9^. This cohort offers an ideal testing ground for evaluating a heterogeneity-based imaging biomarker. While our study is not disease-specific in its scope or aims, we leverage the richness of this dataset to evaluate whether MIRV correlates with established markers of response, including volumetric response heterogeneity, radiological classification, and post-treatment circulating tumor DNA (ctDNA) positivity—a marker of residual disease increasingly studied in metastatic cancers including sarcoma^10–13^.

By examining MIRV’s association with clinical and molecular response metrics, we aim to assess its potential role as an image-based biomarker of treatment response. We further explore whether MIRV holds prognostic value in select patient subsets. Together, these analyses establish MIRV as a reproducible, interpretable, and readily applicable measure of intertumor heterogeneity that may inform personalized treatment strategies in the metastatic setting.

## Results

**Fig. 1:**
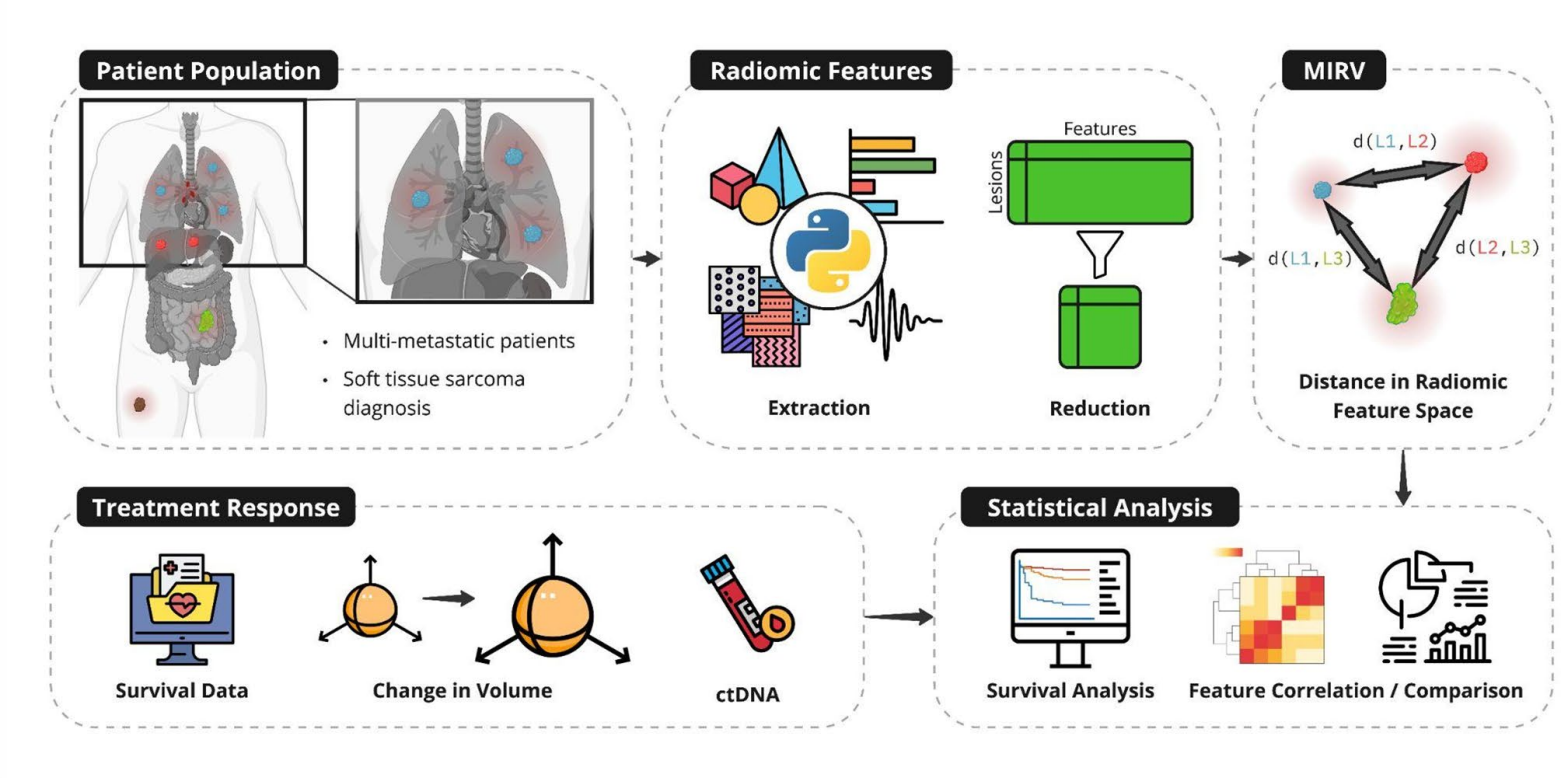
Evaluating intertumor heterogeneity using MIRV as a biomarker of treatment response and clinical covariates. Multi-metastatic STS patients were analyzed (top left). Radiomic features were extracted from baseline imaging and reduced to capture non-redundant tumor-specific characteristics (top center). Intertumor heterogeneity was quantified using MIRV, defined by distances between tumors in radiomic feature space (top right). Treatment response metrics, including survival data, tumor-specific volumetric changes, and ctDNA positivity, were collected (bottom left). Statistical analyses were performed to correlate MIRV with treatment outcomes, including survival analysis and feature comparison (bottom right).

This study introduces MIRV as a novel metric to quantify intertumor heterogeneity using standard-of-care imaging. We applied MIRV to a cohort of multi-metastatic STS patients, extracting and reducing radiomic features from pretreatment CT scans. We then assessed MIRV’s association with treatment response metrics—including volumetric response, tumor-specific response classification (TSRC), and ctDNA positivity—and evaluated its prognostic relevance through statistical analyses (Fig. 1).

### Patient cohort and data collection

We retrospectively analyzed a cohort of metastatic STS patients from SARC021, a phase III clinical trial (TH-CR-406/SARC021, NCT01440088) conducted by the Sarcoma Alliance for Research through Collaboration (SARC)^14^. The study included patients with locally advanced, unresectable, or metastatic disease. To assess MIRV, we focused on patients with multiple metastases contoured on pre-treatment computed tomography (CT) scans. Data collection was approved by the University Health Network institutional ethics review board (REB #20-5707), and the requirement for informed consent was waived due to the retrospective nature of the study. All methods were performed in accordance with the relevant guidelines and regulations. Full trial details can be found in^9^.

The dataset comprised 397 patients, including individuals diagnosed with leiomyosarcoma (41%), liposarcoma (14%), undifferentiated pleomorphic sarcoma (12%), and other STS subtypes (33%). For all patients, we collected pretreatment CT scans and extracted radiomic features alongside clinical characteristics such as RECIST 1.1 assessments and histological subtypes (Table 1). A separate study, independent of SARC021, assessed the feasibility of detecting ctDNA in a subset of the leiomyosarcoma patients^10^, which we were able to incorporate into our analysis. For this subset of patients, ctDNA status was recorded as either positive or negative based on the presence or absence of detectable ctDNA levels, assessed using ichorCNA technology^10^. Additionally, for patients with pulmonary metastases, we examined post-treatment CT scans to evaluate volumetric response. TSRC was applied using volumetric measurements for the lung subset, while RECIST-based response was assessed for the full cohort.

**Table 1:**
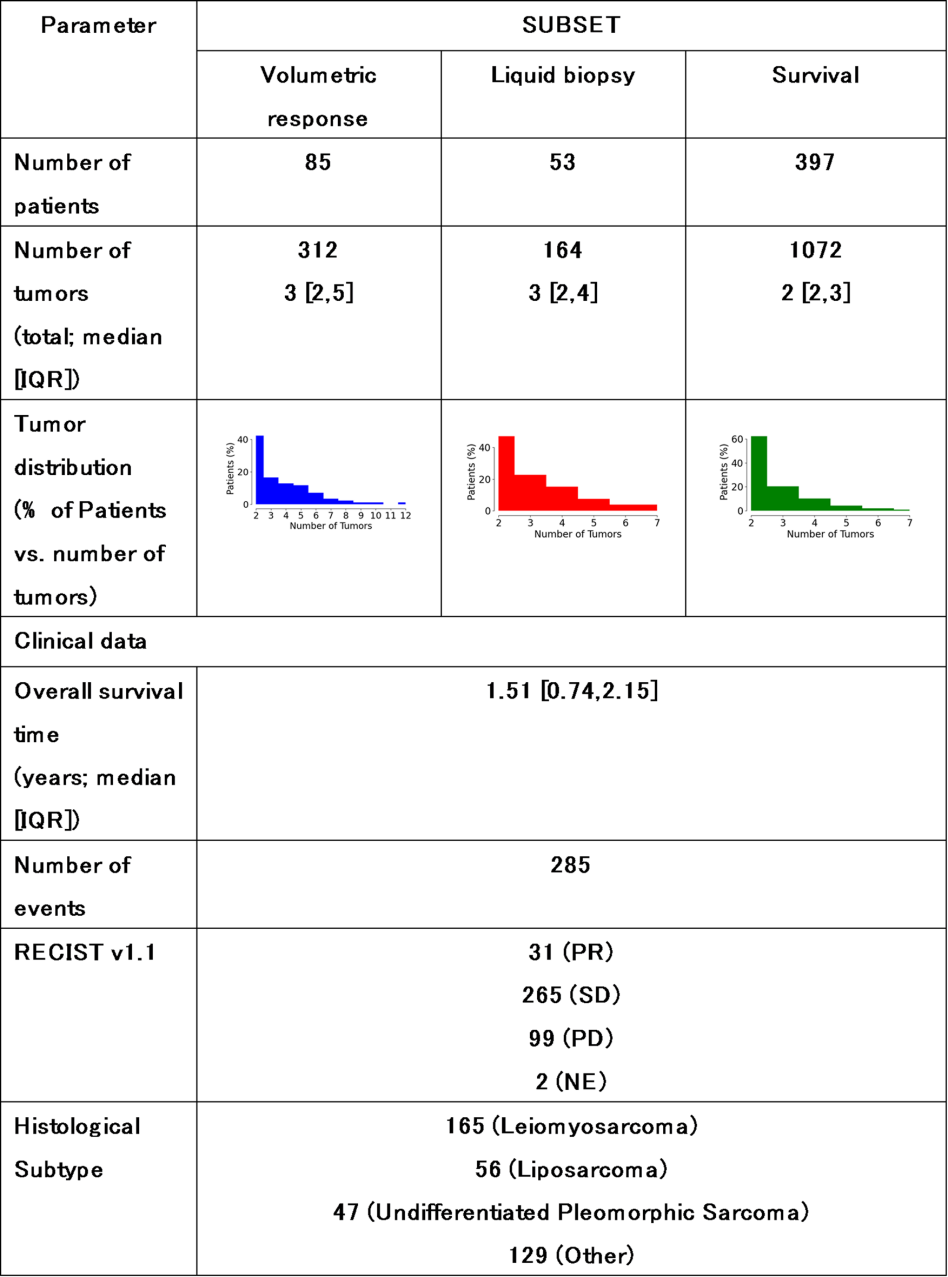
Demographics and clinical information for the dataset used in this study.

Statistics surrounding the number of tumors per patient and survival are expressed in terms of the median and the interquartile range (IQR).

### MIRV and response metrics

Radiomic feature extraction was performed on CT images using PyRadiomics (version 3.0.1)^15^. To reduce redundancy, a stepwise feature reduction process was applied, removing low-variance features, those correlated with tumor volume (|ρ| > 0.1), and highly correlated features (|ρ| > 0.7)^16^. The remaining features were used to compute MIRV metrics (Supplementary Tables S1 and S2). MIRV was then quantified using pairwise cosine dissimilarity and Euclidean distance between tumor radiomic feature vectors within each patient. Cosine dissimilarity (1 − cosine similarity) captures differences in feature orientation^17^, reflecting whether tumors share similar radiomic patterns regardless of absolute feature values. Euclidean distance measures the overall difference between feature vectors, incorporating both magnitude and direction^17^. Together, these metrics provide a comprehensive assessment of intertumor heterogeneity.

We evaluated the relationship between MIRV and overall survival (OS), volumetric response, TSRC, and ctDNA positivity (Table 2). Survival serves as a definitive clinical endpoint, capturing long-term patient outcomes; however, it can be influenced by undocumented factors, making attribution to tumor heterogeneity challenging. Volumetric response measures differences in tumor volume change over time, providing an objective assessment of treatment effects across lesions. While sensitive to response heterogeneity, it may not fully capture non-size-based treatment effects. TSRC categorizes whether all tumors in a patient met a predefined 33% volume reduction threshold^18^, offering a simplified binary measure of response consistency. However, it does not account for partial responses or variations in response magnitude. Finally, ctDNA positivity from liquid biopsy analysis may serve as a non-invasive indicator of tumor burden and tumor response to therapy. While valuable, ctDNA detection may be influenced by tumor shedding dynamics and assay sensitivity^19,20^. Additional details are provided in the Methods section.

**Table 2:** Summary of MIRV and response metrics, including OS, volumetric response, TSRC and liquid biopsy data.

Category	Metric	Description	Measurement	Notes
MIRV	MIRV (max) Distance	Maximum Euclidean Distance evaluated across all possible tumor combinations	Continuous	Calculated using (1)
	MIRV (max) Dissimilarity	Maximum Cosine Dissimilarity evaluated across all possible tumor combinations	Continuous	Calculated using (2)
Response	OS	Right-censored OS data, where the event is patient death from any cause	Continuous, censored	Clinical data
	Volumetric Response	Range of tumor- specific volume change between baseline and first follow-up	Continuous	Calculated using PyRadiomics VoxelVolume feature
	TSRC	Indicator whether all tumors (1), or at least one tumor (0) did not exceed the response threshold	Binary [0,1]	Tumor-specific response threshold set to ΔV > 33%
	ctDNA Positivity	Detectable levels of ctDNA in sarcoma dataset	Binary [+,-]	Assessed using ichorCNA technology; linked to minimal residual disease

### Association between MIRV, tumor response, and liquid biopsy markers

**Fig. 2:**
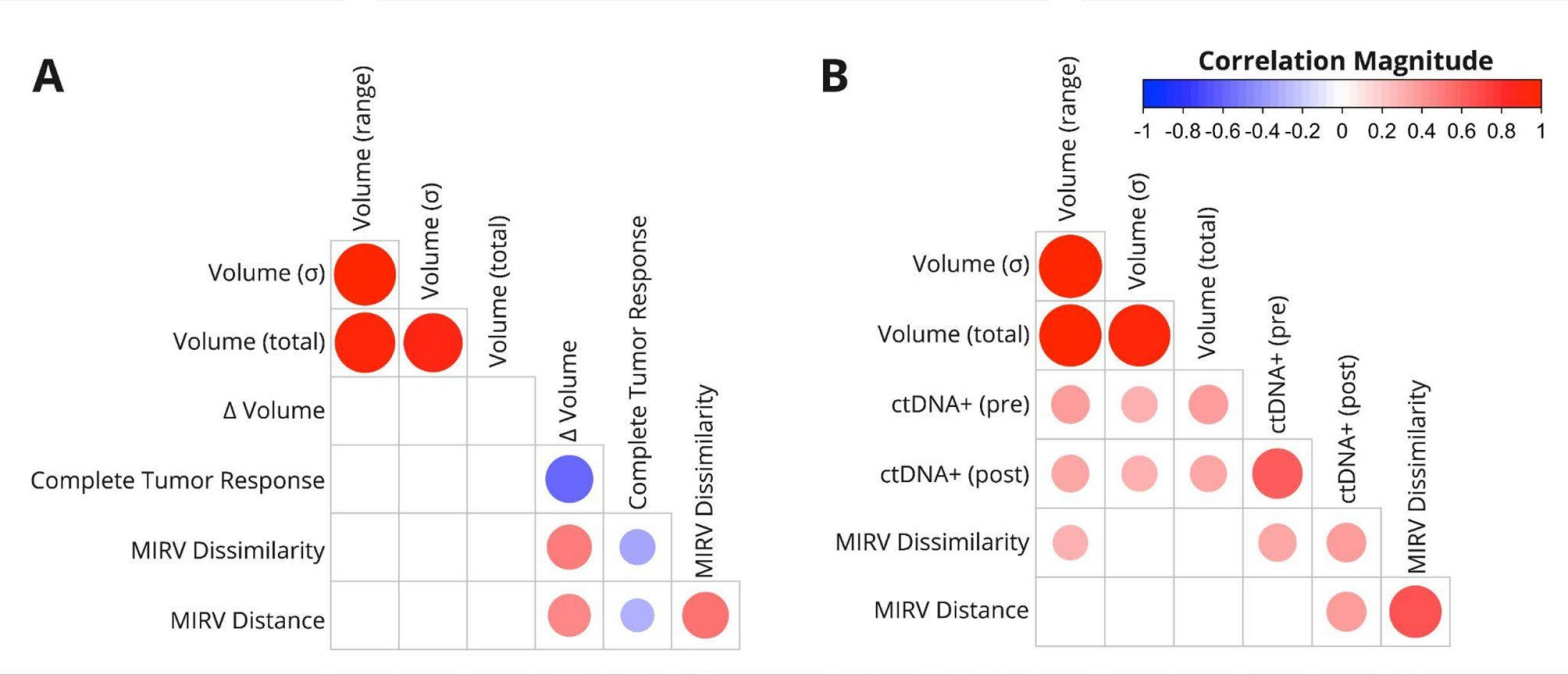
Correlation between MIRV, tumor response, and ctDNA positivity. (A) Relationships between MIRV, TSRC (Complete Tumor Response), and volumetric changes in the volumetric response subset. (B) Relationships between MIRV, and ctDNA pre- and post-treatment in the liquid biopsy subset. Baseline tumor volume characteristics were included as a control. Circle size represents correlation strength (FDR), with colors indicating Spearman’s correlation coefficients (ρ) according to the scale bar. In cases where the FDR exceeded 5%, circles are not displayed.

Our analysis revealed significant associations between MIRV, TSRC, volumetric response, and ctDNA positivity (Fig. 2). Both MIRV metrics exhibited a strong negative correlation with Complete Tumor Response, suggesting that greater intertumor heterogeneity may be linked to non-uniform tumor responses within individual patients. Additionally, MIRV demonstrated a moderate correlation with volumetric response, independent of baseline tumor volume, reinforcing that radiomic heterogeneity captures treatment-related changes beyond tumor burden alone. This distinction is critical, as prior studies have shown that radiomic features can act as surrogates for volume when predicting survival^16,21,22^. To further validate that MIRV was not simply a volume-driven metric, we evaluated its correlation with three baseline volume measures (total tumor volume, range, and standard deviation of tumor volumes). As shown in Fig. 2A, MIRV Distance exhibited minimal correlation with these baseline volume metrics, supporting that MIRV captures biologically meaningful heterogeneity beyond size effects.

In the liquid biopsy subset (Fig. 2B), MIRV metrics showed a moderate correlation with post-treatment ctDNA positivity. Given that ctDNA reflects tumor burden^11,12^, its association with baseline volume metrics was expected. However, MIRV Distance remained largely independent of baseline tumor volume, reinforcing that intertumor heterogeneity captures intrinsic tumor biology beyond size alone. The differing correlation patterns observed between MIRV (cosine vs. Euclidean distance) and baseline volume measures suggest that these metrics may capture complementary aspects of radiomic heterogeneity, with cosine distance being more sensitive to differences in feature composition rather than absolute magnitude. These findings highlight MIRV’s potential as a biomarker linking radiological and molecular indicators of treatment response.

Sensitivity analyses further confirmed that associations between MIRV and key response outcomes were consistent across alternative MIRV definitions (Supplementary Table S3), supporting the robustness of MIRV as a biomarker of intertumor heterogeneity. To further assess the clinical relevance of MIRV, we performed comparative ROC analyses for two clinically relevant binary endpoints: tumor-specific response classification (TSRC) and post-treatment ctDNA positivity. Models incorporating MIRV alongside baseline clinical variables and RECIST response showed improved discrimination compared to baseline or RECIST-only models. In the volumetric subset (TSRC prediction, *n* = 85), the baseline model achieved an AUC of 0.70 ± 0.11, which increased to 0.74 ± 0.10 with the addition of MIRV and 0.75 ± 0.12 when both MIRV and RECIST were included. In the ctDNA subset (*n* = 53), AUC improved from 0.66 ± 0.21 (baseline) to 0.77 ± 0.12 with MIRV alone, and to 0.79 ± 0.10 in the full model (Supplementary Table S5). Although pairwise differences in AUC between nested models were not statistically significant (Z-tests, all *p* > 0.05; Supplementary Table S6), this was expected given the modest sample sizes and high correlation between models^23,24^. Nevertheless, the observed trend toward increased mean AUC with the inclusion of MIRV suggests potential incremental discriminative value across both endpoints. (Supplementary Figure S1).

### MIRV exhibits context-specific survival implications

**Fig. 3:**
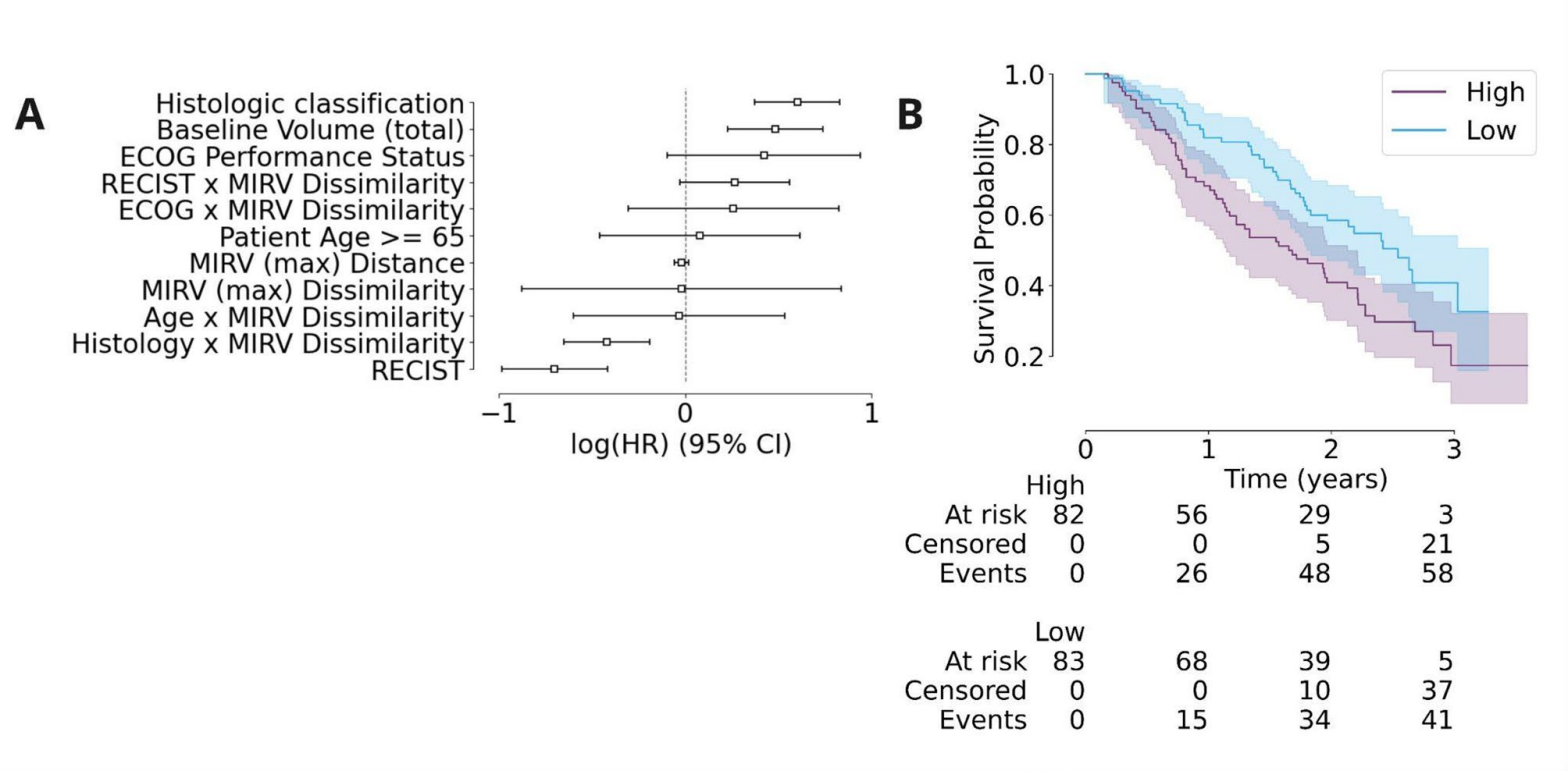
(A) Forest plot showing the log(HR) and 95% confidence intervals for multiple clinical and imaging-based covariates, including MIRV (max) Dissimilarity and Distance. (B) Kaplan-Meier survival curves stratified by MIRV Dissimilarity, with high MIRV (blue) associated with worse survival probability compared to low MIRV (orange). Shaded areas represent the 95% confidence intervals.

We used a multivariable Cox proportional hazards model to assess the prognostic significance of MIRV while controlling for known clinical factors, namely histologic classification, observed baseline volume, performance status, patient age and RECIST classification. This approach allowed us to determine whether MIRV provided independent prognostic information beyond established clinical variables. After adjusting for these factors, no significant association between MIRV and OS was observed in the full cohort. However, subgroup analysis was conducted based on a significant interaction term between MIRV and histological subtype identified in the multivariable model (Fig. 3A, Supplementary Table S4). In these subgroup analyses, log-rank tests revealed that higher MIRV was associated with worse overall survival in the leiomyosarcoma cohort (*n* = 165) with a nominal p-value = 0.007 | FDR = 6% and nominal p-value = 0.06 | FDR = 24% for MIRV Dissimilarity (Fig. 3B) and for MIRV Distance, respectively. These findings suggest that, while MIRV does not have prognostic significance across the full cohort, it may have potential prognostic relevance in specific subgroups such as leiomyosarcoma, which warrants further investigation.

## Discussion

Our study introduces MIRV as a novel imaging-based biomarker for assessing tumor heterogeneity and treatment response. While MIRV was not significantly associated with survival across the entire cohort, sarcomas are highly heterogeneous. In histologic subgroup analysis, MIRV showed potential prognostic relevance, particularly in leiomyosarcoma. The LMS subgroup finding emerged from an exploratory post hoc analysis prompted by a significant MIRV-by-histology interaction term. To account for multiple testing across the four histologic subgroups, we applied false discovery rate (FDR) correction. The association in LMS yielded a FDR = 6%. While this slightly exceeds the conventional 5% threshold, it reflects a low expected proportion of false positives among the discoveries and suggests a potentially meaningful signal. We therefore interpret this result as preliminary but promising. Further validation in independent LMS cohorts will be needed to confirm its clinical and biological relevance. Notably, MIRV exhibited complementary associations with clinically relevant response metrics, including TSRC, tumor volume changes, and ctDNA positivity, highlighting its ability to capture distinct aspects of treatment response. Given the complexity of survival as an outcome measure, these findings suggest that MIRV provides independent and biologically meaningful insights into tumor heterogeneity that may inform prognosis.

Traditional radiomic features have been proposed as descriptors of tumor heterogeneity, but many primarily act as surrogates for baseline tumor volume^16,22^. Our approach extends radiomics beyond the conventional framework—where typically only one tumor per patient is analyzed^25^ —to a paradigm that incorporates multiple tumors per patient. This enables a more comprehensive assessment of tumor heterogeneity and its relationship to treatment response, independent of baseline tumor burden. Consistent with this, MIRV metrics were derived from a feature set explicitly de-correlated from tumor volume (|ρ| > 0.1 exclusion), and subsequent validation demonstrated that MIRV Distance exhibited minimal correlation with baseline volume metrics. We selected maximum pairwise distance as the primary MIRV definition to capture the most extreme divergence in radiomic phenotype across a patient’s lesions, based on the rationale that even a single highly divergent or resistant lesion may disproportionately impact clinical outcomes such as treatment failure or progression. To further support the robustness of this metric, sensitivity analyses demonstrated that associations with key clinical outcomes were consistent across alternative MIRV definitions (mean, median, and standard deviation of pairwise distances). Furthermore, MIRV is evaluated on a per-patient basis, mitigating image-related biases common in multi-institutional studies caused by variations in imaging protocols and equipment^26^. This normalization enhances MIRV’s applicability across diverse patient populations.

MIRV metrics showed a moderate correlation with post-treatment ctDNA positivity. Given that ctDNA reflects tumor burden^11,12^, its association with baseline volume metrics was expected. However, MIRV Distance remained largely independent of baseline tumor volume, reinforcing that intertumor heterogeneity captures intrinsic tumor biology beyond size alone. These findings suggest that patients with higher intertumor heterogeneity may be more likely to exhibit treatment-resistant or persistent lesions, contributing to residual ctDNA. Importantly, the observed association between MIRV and post-treatment ctDNA positivity was not explained by total tumor burden, as MIRV Distance was minimally correlated with baseline tumor volume metrics (Fig. 2). In this study, ctDNA positivity was defined consistent with prior work^10^, using the ichorCNA threshold of 3% as the validated limit of detection. In response to reviewer feedback, we also explored potential associations between MIRV and changes in ctDNA positivity (pre- to post-treatment). No significant association was observed in the current dataset. However, given the limitations of using a binarized metric to capture ctDNA dynamics, further investigation with larger cohorts and continuous molecular measures will be important to fully elucidate this relationship.

The clinical implications of our findings highlight MIRV’s potential to supplement existing treatment response metrics, offering a new dimension for oncological decision-making. These findings suggest that MIRV could serve as a complementary biomarker for stratifying patients at risk of heterogeneous treatment response or molecular persistence of disease. Exploratory ROC analyses support this potential, showing that MIRV improves model performance for both radiological and molecular endpoints (TSRC and post-treatment ctDNA positivity) when added to baseline clinical features and RECIST response. While pairwise differences in AUC between nested models did not reach statistical significance—likely due to limited sample sizes and high correlation between predictors—the consistency of improvement across models supports the hypothesis that MIRV captures clinically relevant intertumor heterogeneity. These results motivate further evaluation of MIRV in larger, prospective cohorts designed to assess treatment adaptation or escalation in patients with high intrapatient heterogeneity.

To further validate its utility, prospective studies in other cancer types and investigations into its predictive power for targeted therapies, such as immunotherapy and radiation therapy, are warranted. Despite these promising findings, our study has limitations. The retrospective design and dataset-specific biases may limit generalizability, and additional research is needed to optimize MIRV thresholds for clinical use. Another limitation is the omission of tumor grade from the multivariable survival model. While tumor grade is a well-established prognostic factor in soft tissue sarcoma, the grading system was not consistently applied across the SARC021 cohort, limiting its interpretability and justifying its exclusion in this context. A key challenge is the scarcity of datasets containing sequential volumetric segmentations, essential for tracking tumor kinetics over time. However, our MIRV analysis pipeline is openly available, and we encourage researchers to apply it to their own datasets or collaborate on future research to expand its clinical relevance.

## Conclusion

Our study demonstrates that MIRV provides unique insights into tumor biology, heterogeneity, and treatment response, beyond traditional prognostic markers. By capturing tumor-specific variability, MIRV may enhance patient stratification and guide more personalized therapeutic strategies. Integrating MIRV into clinical workflows could refine response assessment and improve precision oncology. While MIRV’s direct prognostic utility remains to be fully established, its role in characterizing treatment-related heterogeneity suggests a potential avenue for further research and clinical translation.

## Methods

### Data processing and analysis

#### Radiomic feature handling

Radiomic feature extraction was performed on CT images using PyRadiomics (version 3.0.1)^15^, with tumor volume defined as the *VoxelVolume* feature for each individual tumor. Tumor segmentations were generated using a semi-automated approach with subsequent radiologist review. To address feature redundancy and mitigate potential confounding by tumor size, we implemented a stepwise feature reduction pipeline prior to MIRV calculation. First, features with low variance (defined as below the median variance across all features) were removed. Next, to explicitly reduce volume-related effects, features demonstrating an absolute Spearman correlation (|ρ| > 0.1) with *VoxelVolume* were excluded^16^. This ensured that MIRV would be derived from features largely independent of tumor size. Finally, any remaining features with high redundancy (|ρ| > 0.7 with any other feature) were removed. The resulting de-correlated feature set was then used to compute MIRV metrics.

#### MIRV metrics

To calculate MIRV metrics, we developed a function to compute pairwise cosine dissimilarity (1 − cosine similarity) and Euclidean distance between tumors for each patient. Cosine dissimilarity captures differences in feature orientation, making it robust to variations in absolute feature magnitudes, while Euclidean distance reflects overall dissimilarity in feature space, accounting for both magnitude and direction. All radiomic features were z-score normalized prior to distance calculation to ensure equal weighting across features when computing Euclidean and cosine distances. Together, these metrics provide complementary insights into intertumor heterogeneity—cosine dissimilarity emphasizing shape-based differences and Euclidean distance capturing absolute divergence in tumor characteristics. For each patient, pairwise metrics were calculated across all possible tumor combinations, and the maximum values were used to summarize intertumor relationships, highlighting the most divergent tumor pair per patient (1, 2).

1 $${\text{MIRV(max}}{{\text{)}}_{{\text{Distance}}}}{\text{=max}}\left( {{{\text{d}}_{{\text{Euclidean}}}}\left( {{{\text{L}}_{\text{i}}}{\text{,}}{{\text{L}}_{\text{j}}}} \right)\forall {\text{i,j}}} \right)$$

2 $${\text{MIRV(max}}{{\text{)}}_{{\text{Dissimilarity}}}}{\text{=max}}\left( {{\text{dissi}}{{\text{m}}_{{\text{Cosine}}}}\left( {{{\text{L}}_{\text{i}}}{\text{,}}{{\text{L}}_{\text{j}}}} \right)\forall {\text{i,j}}} \right)$$

#### Response metrics

Response metrics included OS expressed in years, and volumetric response assessed at a post-treatment timepoint, TSRC and liquid biopsy data (Table 2).

#### Volumetric response

Volumetric response was assessed by calculating the relative change in tumor volume between baseline and first follow-up scans. Contours delineated at both time points were used to perform these calculations. The PyRadiomics **‘VoxelVolume’** feature served as the measure of tumor volume at baseline and follow-up. Relative volume change was expressed as a percentage. At the patient level, the range in volume change across all tumors was assessed and reported as a percentage.

#### TSRC

To assess intertumor variability in treatment response, patients were categorized based on the concordance of volumetric changes across their metastatic tumors. Tumor responses were evaluated using a predefined volume change threshold (ΔV > 33%)^18^. Patients were classified as having Complete Tumor Response if all tumors exhibited a change in volume that did not exceed the threshold. Patients that had at least one tumor that exceeded the response threshold, including cases where all tumors exceeded the threshold, were classified as Non-/Partial Tumor Response.

#### Liquid biopsy data

For this dataset, we use the presence of detectable ctDNA levels as a metric for therapy response, which was recorded as binary data (positive or negative) based on the presence or absence of detectable levels of ctDNA in the sample. The collection and processing of ctDNA data for the sarcoma dataset are described in^10^; briefly, a positive ctDNA test indicates microscopic amounts of cancer cells, often referred to as minimal residual disease (MRD), in the patient’s blood. The levels of detectable ctDNA were assessed using the ichorCNA technology.

### Statistical analysis

We conducted two main types of analyses: (1) correlation between MIRV and treatment response metrics, and (2) survival analysis evaluating MIRV alongside clinical and radiological metrics.

#### Correlation between MIRV and treatment response

Spearman’s rank correlation coefficient was used to evaluate associations between MIRV and treatment response metrics. This non-parametric method was selected due to the presence of non-normally distributed variables and varied data types, providing a robust assessment of monotonic relationships. For the lung metastasis subset, MIRV was correlated with changes in tumor volume and TSRC. For the liquid biopsy subset, MIRV was compared with ctDNA positivity to investigate its relationship with molecular response. Given the potential sensitivity of MIRV to baseline tumor volume, we also considered three baseline volumetric measures: (1) range in tumor volumes (absolute difference between the smallest and largest tumor per patient), (2) standard deviation of tumor volumes, and (3) total tumor volume. Baseline volume metrics were derived using the PyRadiomics VoxelVolume feature, consistent with volumetric response analyses. To account for multiple hypothesis testing while preserving power, p-values were corrected using the Benjamini-Hochberg False Discovery Rate (FDR) method, as implemented in the statsmodels package.

To evaluate the incremental predictive value of MIRV beyond standard clinical and imaging variables, we constructed logistic regression models for two binary endpoints: TSRC (based on volumetric response subset) and post-treatment ctDNA positivity (liquid biopsy subset). Feature sets included combinations of baseline clinical covariates (age ≥ 65, ECOG performance status, histologic classification, baseline tumor volume), RECIST response, and MIRV metrics (MIRV Distance and MIRV Dissimilarity). Categorical variables were encoded as ordinal values.

Models were trained using stratified 5-fold cross-validation, which allows for a fair comparison of predictor sets while minimizing overfitting. This approach is conceptually similar to pre-validation^27^, enabling the evaluation of new predictors (e.g., MIRV) alongside established features within the same dataset without favoring the model containing the newly derived variables. Hyperparameter tuning for regularization strength (C = 10^[− 3, − 2, …, 3]) was conducted using GridSearchCV for logistic regression with L2 penalty (solver=’lbfgs’, max_iter = 1000). Model discrimination was assessed using the mean area under the ROC curve (AUC) across folds. To evaluate performance stability, we report the standard deviation of AUC across folds. ROC curves were generated by interpolating true positive rates across a common false positive rate grid. Pairwise comparisons of AUC values between models were evaluated using Z-tests.

#### Survival analysis

The prognostic value of MIRV was first assessed using continuous MIRV values in a Cox proportional hazards model to evaluate its association with OS. Additionally, we computed the Hazard Ratio to quantify the predictive ability of MIRV relative to clinical covariates, including performance status, patient age, histological subtype, RECIST response, and total tumor burden (measured by total volume). Subgroup analyses were conducted based on interaction terms identified in the multivariable model, with Kaplan-Meier survival analysis and log-rank tests performed to evaluate OS differences within specific patient subgroups. All survival analyses were conducted using the lifelines package in Python.

#### Sensitivity analysis

To assess the robustness of our findings to MIRV definition, we conducted a post hoc sensitivity analysis comparing four MIRV aggregation metrics: maximum, mean, median, and standard deviation of pairwise distances. Spearman correlations with response endpoints (TSRC, volumetric response, ctDNA positivity) and associated FDR were computed for each definition.

## Electronic supplementary material

Below is the link to the electronic supplementary material.

Supplementary Material 1

## Data Availability

Imaging and clinical data for the SARC021 trial are not publicly available but can be obtained through the Sarcoma Alliance for Research through Collaboration (SARC) with a Data Use Agreement (DUA). For more information on accessing data and required documentation, including the Data Access Application Form, please visit the SARC Clinical Data Repository. For questions or to initiate a request, contact the SARC UDS Coordinator at sarc-uds@sarctrials.org. The de-identified radiomic features and clinical information, along with the code for data cleaning and analysis that underlie the results reported in the article are publicly available. The computer code is available from https://github.com/bhklab/MIRV, with relevant parameter files to replicate the software environment used to generate the results in this paper. Notably, the environment for feature extraction and analysis was configured using Pixi, enabling compatibility across Windows, Mac, and Linux operating systems. Additionally, the full software environment, processed data and computer code are available from https://codeocean.com/capsule/8750010/tree/v1.
