# Supplementary material for: Measured intrapatient radiomic variability as a predictor of treatment response in multi-metastatic soft tissue sarcoma patients
Source: Sci Rep. 2025 Jul 30;15:27838. doi: 10.1038/s41598-025-12451-3 (PMC12311145; doi:10.1038/s41598-025-12451-3)
Supplement: Supplementary file 1 — Supplementary Material 1 [file 41598_2025_12451_MOESM1_ESM.pdf]

# Supplemental

| Number of Features | SUBSET | | |
|---|---|---|---|
| | Volumetric Response | Liquid Biopsy | Survival |
| Total Extracted | 1218 | | |
| High Variance, Uncorrelated to Volume | 248 | 265 | 261 |
| High Variance, Uncorrelated to Volume and Other Features | 16 | 10 | 13 |

**Table S1:** *Summary of radiomic features used to calculate MIRV for each subset of the patient data: volumetric response, liquid biopsy and survival. Numbers of features used for the calculation are recorded at different stages of the unsupervised feature reduction step.*

| Radiomics Features | | | SUBSET | | |
|---|---|---|---|---|---|
| Filter | Class | Name | Volumetric Response | Liquid Biopsy | Survival |
| Gradient | GLSZM | SmallAreaEmphasis | ✅ | ✅ | ✅ |
| Wavelet-HHL | GLCM | ClusterShade | ✅ | ✅ | ✅ |
| Wavelet-HHL | FirstOrder | Median | ✅ | | ✅ |
| Wavelet-HHH | FirstOrder | Median | ✅ | | ✅ |
| SquareRoot | GLCM | ClusterShade | ✅ | | ✅ |
| Wavelet-HLH | FirstOrder | Median | ✅ | | ✅ |
| Wavelet-LHH | FirstOrder | Median | ✅ | | ✅ |
| Wavelet-LLH | GLCM | ClusterShade | ✅ | | ✅ |
| Exponential | GLSZM | SmallAreaEmphasis | | ✅ | ✅ |
| Wavelet-HLH | GLSZM | SmallAreaEmphasis | | ✅ | ✅ |
| Wavelet-LHL | GLCM | ClusterShade | | ✅ | ✅ |
| Wavelet-LLL | GLCM | Correlation | | ✅ | ✅ |

| | | | | | |
|---|---|---|---|---|---|
| Wavelet-HLH | GLCM | ClusterShade | ✅ | ✅ | |
| Wavelet-HLL | GLCM | ClusterShade | ✅ | ✅ | |
| Wavelet-LHH | GLCM | ClusterShade | ✅ | ✅ | |
| Wavelet-HLL | FirstOrder | Kurtosis | | ✅ | |
| Wavelet-LLH | GLCM | Correlation | ✅ | | |
| Wavelet-HHH | GLSZM | SmallAreaEmphasis | ✅ | | |
| Square | GLSZM | SmallAreaEmphasis | ✅ | | |
| Logarithm | FirstOrder | InterquartileRange | ✅ | | |
| Logarithm | GLDM | LargeDependenceHigh GrayLevelEmphasis | ✅ | | |
| Wavelet-LHH | GLSZM | SmallAreaEmphasis | | | ✅ |

**Table S2:** *Summary of radiomic features used to calculate MIRV for each subset of the patient data: volumetric response, liquid biopsy and survival. Five features are used in all three subsets, and sixteen features are used by at least two subsets. Five features are unique to the volumetric response subset and three features are unique to the liquid biopsy subset.*

| Subset | Outcome | MIRV Definition | Spearman ρ | FDR |
|---|---|---|---|---|
| Volumetric Response | ΔVolume | Max | 0.51 | 0 |
| | | Mean | 0.24 | 0.06 |
| | | Median | 0.24 | 0.05 |
| | | Std. Dev. | 0.45 | 0 |
| | TSRC | Max | -0.32 | 0.01 |
| | | Mean | -0.18 | 0.15 |
| | | Median | -0.19 | 0.12 |

| | | | | |
|---|---|---|---|---|
| | | Std. Dev. | -0.31 | 0.01 |
| Liquid Biopsy | ctDNA (pre) | Max | 0.28 | 0.07 |
| | | Mean | 0.07 | 0.72 |
| | | Median | 0.09 | 0.63 |
| | | Std. Dev. | 0.41 | 0.01 |
| | ctDNA (post) | Max | 0.36 | 0.02 |
| | | Mean | 0.30 | 0.05 |
| | | Median | 0.24 | 0.12 |
| | | Std. Dev. | 0.26 | 0.09 |

**Table S3:** *Sensitivity analysis of MIRV definition. Values are shown for MIRV Dissiilarity. Associations between four aggregation metrics (maximum, mean, median, standard deviation of pairwise distances) and key treatment response metrics. Spearman's rank correlation coefficient ([illegible]) and FDR-adjusted p-values are reported. Consistent associations were observed across MIRV definitions, supporting the robustness of MIRV as a biomarker of intertumor heterogeneity.*

| Variable | **Hazard Ratio [95% conf.int.]** | **z** | **p** |
|---|---|---|---|
| Patient Age >= 65 | 0.88 [0.50,1.56] | -0.43 | 0.67 |
| ECOG Performance Status | 1.46 [0.84,2.53] | 1.35 | 0.18 |
| Histologic classification | 1.82 [1.44,2.31] | 4.94 | <0.005 |
| MIRV (max) Dissimilarity | 1.21 [0.62,2.38] | 0.55 | 0.58 |
| MIRV (max) Distance | 1.00 [0.96,1.05] | 0.07 | 0.94 |
| Baseline Volume (total) | 1.57 [1.21,2.05] | 3.35 | <0.005 |
| RECIST | 0.54 [0.40,0.72] | -4.08 | <0.005 |
| ECOG x MIRV Dissimilarity | 1.26 [0.68,2.37] | 0.73 | 0.46 |
| Histology x MIRV Dissimilarity | 0.68 [0.52,0.87] | -3.04 | <0.005 |

| | | | |
|---|---|---|---|
| Age x MIRV Dissimilarity | 1.18 [0.63,2.21] | 0.51 | 0.61 |
| RECIST x MIRV Dissimilarity | 1.14 [0.82,1.59] | 0.79 | 0.43 |

**Table S4:** *Hazard ratios (HR) with 95% confidence intervals (CI), z-scores, and p-values for key clinical and radiomic variables in a multivariable Cox proportional hazards model assessing OS in metastatic soft-tissue sarcoma patients. Significant predictors of worse survival include histologic classification (HR = 1.82, p < 0.005), and baseline tumor volume (HR = 1.57, p < 0.005). RECIST response was significantly associated with better survival (HR = 0.54, p < 0.005). MIRV-based metrics (dissimilarity and distance) were not significant prognostic factors (p = 0.58 and p = 0.94, respectively), but the histology-MIRV interaction term (Dissimilarity) was significantly associated with better survival (HR = 0.68, p < 0.005).*

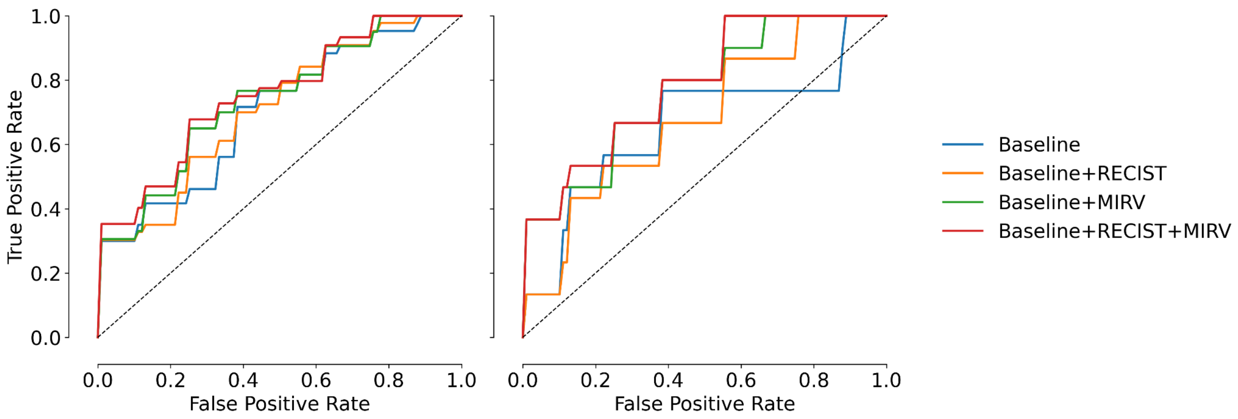


**Supplementary Figure S1:** *Comparative ROC curves evaluating model performance for prediction of (Left) tumor-specific radiological response classification (TSRC) in the volumetric response subset and (Right) post-treatment ctDNA positivity in the liquid biopsy subset. Logistic regression models were trained using baseline clinical features (age ≥ 65, ECOG performance status, histologic classification, baseline tumor volume), with incremental inclusion of RECIST response and MIRV metrics (Distance and Dissimilarity). ROC curves represent the mean true positive rate across five stratified folds.*

| Outcome | Model | AUC (mean ± SD) |
|---|---|---|
| TSRC (Volumetric subset) | Baseline | 0.70 ± 0.11 |
| | Baseline + RECIST | 0.71 ± 0.10 |
| | Baseline + MIRV | 0.74 ± 0.10 |
| | Baseline + RECIST + MIRV | 0.75 ± 0.12 |
| Post-treatment ctDNA Positivity (ctDNA subset) | Baseline | 0.66 ± 0.21 |
| | Baseline + RECIST | 0.68 ± 0.15 |

| | | |
|---|---|---|
| | Baseline + MIRV | 0.77 ± 0.12 |
| | Baseline + RECIST + MIRV | 0.79 ± 0.10 |

**Supplementary Table S5:** *Area under the ROC curve (AUC) with standard deviation for logistic regression models predicting (top) TSRC in the volumetric subset and (bottom) post-treatment ctDNA positivity in the liquid biopsy subset. Models included baseline clinical covariates (age ≥ 65, ECOG performance status, histology, and total baseline tumor volume), with incremental inclusion of RECIST response and MIRV metrics. AUCs were computed via 5-fold stratified cross-validation.*

| Subset | Comparison | Z-Score | P-Value |
|---|---|---|---|
| TSRC (Volumetric subset) | Baseline vs Baseline + RECIST | -1.63 | 0.103 |
| | Baseline vs Baseline + MIRV | -0.83 | 0.405 |
| | Baseline vs Baseline + RECIST + MIRV | -1.59 | 0.112 |
| | Baseline + RECIST vs Baseline + MIRV | -0.11 | 0.911 |
| | Baseline + RECIST vs Baseline + RECIST + MIRV | -0.91 | 0.361 |
| | Baseline + MIRV vs Baseline + RECIST + MIRV | -1.10 | 0.269 |
| Post-treatment ctDNA Positivity (ctDNA subset) | Baseline vs Baseline + RECIST | -0.29 | 0.765 |
| | Baseline vs Baseline + MIRV | -1.44 | 0.148 |
| | Baseline vs Baseline + RECIST + MIRV | -1.38 | 0.167 |
| | Baseline + RECIST vs Baseline + MIRV | -1.87 | 0.061 |
| | Baseline + RECIST vs Baseline + RECIST + MIRV | -1.70 | 0.089 |

| | | | |
|---|---|---|---|
| | Baseline + MIRV vs Baseline + RECIST + MIRV | -0.41 | 0.679 |

**Supplementary Table S6:** *Pairwise comparisons of area under the ROC curve (AUC) between logistic regression models using DeLong Z-tests. Comparisons were made across models incorporating baseline clinical covariates, RECIST response, and MIRV metrics. While several model comparisons showed trends toward improved performance with MIRV, none reached statistical significance (all p > 0.05). Subsets correspond to tumor-specific response classification (TSRC, lung subset) and post-treatment ctDNA positivity (liquid biopsy subset).*
